# The Core Promoter Is a Regulatory Hub for Developmental Gene Expression

**DOI:** 10.3389/fcell.2021.666508

**Published:** 2021-09-10

**Authors:** Anna Sloutskin, Hila Shir-Shapira, Richard N. Freiman, Tamar Juven-Gershon

**Affiliations:** ^1^The Mina and Everard Goodman Faculty of Life Sciences, Bar-Ilan University, Ramat Gan, Israel; ^2^Department of Molecular Biology, Cell Biology and Biochemistry, Brown University, Providence, RI, United States

**Keywords:** development, transcriptional regulation, core promoter, core promoter elements/motifs, Hox genes, dorsal-ventral axis, mesoderm formation, basal transcription machinery

## Abstract

The development of multicellular organisms and the uniqueness of each cell are achieved by distinct transcriptional programs. Multiple processes that regulate gene expression converge at the core promoter region, an 80 bp region that directs accurate transcription initiation by RNA polymerase II (Pol II). In recent years, it has become apparent that the core promoter region is not a passive DNA component, but rather an active regulatory module of transcriptional programs. Distinct core promoter compositions were demonstrated to result in different transcriptional outputs. In this mini-review, we focus on the role of the core promoter, particularly its downstream region, as the regulatory hub for developmental genes. The downstream core promoter element (DPE) was implicated in the control of evolutionarily conserved developmental gene regulatory networks (GRNs) governing body plan in both the anterior-posterior and dorsal-ventral axes. Notably, the composition of the basal transcription machinery is not universal, but rather promoter-dependent, highlighting the importance of specialized transcription complexes and their core promoter target sequences as key hubs that drive embryonic development, differentiation and morphogenesis across metazoan species. The extent of transcriptional activation by a specific enhancer is dependent on its compatibility with the relevant core promoter. The core promoter content also regulates transcription burst size. Overall, while for many years it was thought that the specificity of gene expression is primarily determined by enhancers, it is now clear that the core promoter region comprises an important regulatory module in the intricate networks of developmental gene expression.

## Introduction

The uniqueness of each cell type and its various developmental roles in multicellular organisms are largely achieved by distinct transcriptional programs. Transcription initiation is composed of a series of highly conserved, coordinated and complex steps (reviewed in Thomas and Chiang, 2006; Fuda et al., 2009; Danino et al., 2015; Koster et al., 2015). The core promoter region encompasses the ± 40 bp region relative to the transcription start site (TSS; defined as the + 1 or first ribonucleotide of a transcript). This critical regulatory region, which is required for accurate initiation of transcription by Pol II, is often referred to as the “gateway to transcription” (Heintzman and Ren, 2007; Juven-Gershon and Kadonaga, 2010). Since transcriptional regulation by Pol II determines proper organismal growth and development, alterations in transcriptional regulation may result in a variety of phenotypes, including cell fate changes and lethality (Levine and Tjian, 2003; Ohler and Wassarman, 2010; Bolt and Duboule, 2020). Genome-wide analysis of mammalian promoters led to the discovery that transcription initiation may occur in two distinct modes, namely focused and dispersed, with single or multiple start sites, respectively (Carninci et al., 2006). Recent studies suggest that transcription initiation is more intricate, likely involving combinations between the “focused” and “dispersed” modes (Kadonaga, 2012; Lenhard et al., 2012; Frith and Fantom Consortium, 2014). *Drosophila* was instrumental in deciphering the identity and function of core promoter composition. The transcription machinery, as well as its regulatory principles, are largely conserved in evolution. Thus, although most of the data discussed here is largely based on findings in *Drosophila*, many of the underlying principles are applicable to a wider array of species.

Importantly, the regulation of transcription initiation by Pol II occurs at both the DNA (promoter) and the protein (basal transcription machinery) levels. Recent reviews focused on the structure of polymerases, the components of the general/basal transcription machinery and the preinitiation complex (Cramer, 2019a,b; Roeder, 2019; Schier and Taatjes, 2020). In this mini-review, we will highlight the function of the core promoter as the regulatory hub that recruits specific transcription factors to control key developmental gene expression programs.

## Core Promoter Composition

**FIGURE 1:**
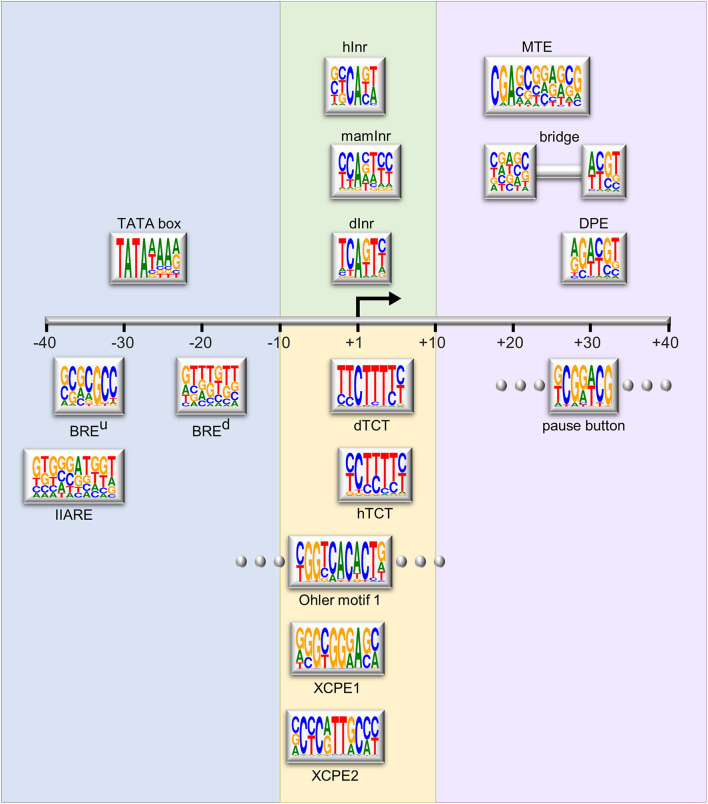
Schematic representation of the major core promoter elements. The region of the core promoter area (–40 to +40 relative to the TSS) is illustrated to scale. The Logo representation for each element is based on the position weight matrix (PWM) derived from functional sequences (Sloutskin et al., 2015), or as provided in the primary reference. Logos were generated using WebLogo (Crooks et al., 2004). Rectangles with flanking dots represent elements that are not strictly spaced. Background colors represent the grouping of the elements based on their position relative to the A_+__1_ or functional similarity. The depicted elements are: upstream and downstream TFIIB recognition elements (BRE^u^ and BRE^d^, respectively, Lagrange et al., 1998; Deng and Roberts, 2005); TFIIA recognition element (IIARE, Wang et al., 2017); TATA box (Goldberg, 1979); *Drosophila* initiator (dInr, Lo and Smale, 1996); mammalian initiator (mamInr, Smale and Baltimore, 1989); focused human initiator (hInr, Vo Ngoc et al., 2017a); *Drosophila* and human polypyrimidine transcription initiation (dTCT and hTCT, respectively, Parry et al., 2010); Ohler motif 1 (Ohler et al., 2002); X core promoter element 1 and 2 (XCPE1 and XCPE2, respectively, Tokusumi et al., 2007; Anish et al., 2009); downstream core promoter element (DPE, Burke and Kadonaga, 1996, 1997; Kutach and Kadonaga, 2000); motif ten element (MTE, Ohler et al., 2002; Lim et al., 2004); bridge (Theisen et al., 2010); pause button (PB, Hendrix et al., 2008).

Although the core promoter was previously considered a universal component that works similarly for all protein-coding genes, it is now established that core promoters are divergent in their architecture and function, and each core promoter is rather unique (Sandelin et al., 2007; Muller and Tora, 2014; Roy and Singer, 2015). The core promoter may contain one or more short DNA sequences, termed core promoter elements or motifs, which contribute to its function. The major sequence elements identified in *Drosophila* and human focused promoters are illustrated in Figure 1 and reviewed in detail in Danino et al. (2015); Vo Ngoc et al. (2017b), and Haberle and Stark (2018).

Importantly, the core promoter serves as the scaffold for the assembly of the pre-initiation complex (PIC), which is comprised of Pol II, the basal transcription factors (TFIIA, TFIIB, TFIID, TFIIE, TFIIF, and TFIIH) and promoter DNA. Multiple core promoter elements were identified as the regions bound by distinct PIC components. The upstream and downstream TFIIB recognition elements (BRE^u^ and BRE^d^, respectively) are bound by TFIIB (Lagrange et al., 1998; Deng and Roberts, 2005). The TFIIA recognition element (IIARE) was recently described (Wang et al., 2017; Wang J. et al., 2020).

The DPE, motif ten element (MTE) and bridge downstream core promoter elements (see below) serve as docking sites for TFIID, which initiates PIC formation by binding to the core promoter. TFIID is composed of TATA box-binding protein (TBP) and 13–14 TBP-associated factors (TAFs) (recently reviewed in Antonova et al., 2019; Bhuiyan and Timmers, 2019; Patel et al., 2020). The first identified and perhaps most well-known core promoter element is the TATA box (Goldberg, 1979), bound by TBP and originally discovered in *Drosophila* histone genes. Both the TATA box and TBP are conserved from archaea to humans (Reeve, 2003). Core promoters were previously classified as having or lacking a TATA-box, yet only a minority of metazoan promoters contain a TATA-box (Gershenzon and Ioshikhes, 2005; Kim et al., 2005; Dikstein, 2011). Thus, TATA-less promoters still require TFIID binding presumably through other core promoter elements.

The initiator (Inr) element, which encompasses the TSS (Corden et al., 1980; Smale and Baltimore, 1989), is the most prevalent core promoter motif within *Drosophila* focused promoters (FitzGerald et al., 2006; Gershenzon et al., 2006). It is bound by the TAF1 and TAF2 subunits of TFIID (Kaufmann and Smale, 1994; Verrijzer et al., 1995; Chalkley and Verrijzer, 1999; Wu et al., 2001; Louder et al., 2016). Functional initiator motifs were first defined in *Drosophila* and mammalian species, and more recently in focused human promoters (Vo Ngoc et al., 2017a). The TCT is a polypyrimidine transcription initiation motif that is conserved from *Drosophila* to humans, enriched in ribosomal protein genes and proteins involved in translational regulation (Hariharan and Perry, 1990; Perry, 2005; Parry et al., 2010). This example highlights the importance of specific core promoter elements for distinct functional transcriptional systems. The X core promoter element 1 and 2 (XCPE1 and XCPE2, respectively), identified around the TSSs of the hepatitis B virus X gene, regulate transcription of a minority of human Pol II promoters (Tokusumi et al., 2007; Anish et al., 2009). While most of the above mentioned core promoter elements are associated with focused promoters, several core promoter motifs are associated with dispersed initiation (Rach et al., 2009; Hoskins et al., 2011). Specifically, Ohler motifs 1, 6, and 7 were computationally identified as over-represented motifs in *Drosophila* core promoters (Ohler et al., 2002). Motif 1 binding protein (M1BP) specifically binds Ohler motif 1, located in the vicinity of the TSS, was identified and biochemically characterized (Li and Gilmour, 2013), providing the experimental validation of the original predictions. Another element associated with dispersed initiation is the DNA-replication-related element (DRE), which was discovered along with its protein binding factor, DREF (Hirose et al., 1993). Notably, a universal core promoter element combination does not exist and novel core promoter motifs are still being discovered.

The DPE motif is the most well characterized downstream motif, precisely located at + 28 to + 33 relative to the A_+__1_ of the Inr (Burke and Kadonaga, 1996, 1997; Kutach and Kadonaga, 2000). The MTE was identified as an overrepresented core promoter sequence located immediately upstream of the DPE (positions + 18 to + 29; Ohler et al., 2002), and was experimentally characterized as a functional regulatory element (Lim et al., 2004). The MTE and DPE partially overlap, and together encompass three functional sub-regions located at nucleotides + 18 to + 22, + 27 to + 29 and + 30 to + 33 downstream of the A_+__1_ (Theisen et al., 2010). The bridge motif was defined as a combination of only the first and third functional sub-regions (bridge I and II, positions + 18 to + 22 and + 30 to + 33, respectively). It is a rare yet functional, core promoter element. Interestingly, recent reviews present the MTE as a bipartite element, replacing the originally identified bridge motif (Vo Ngoc et al., 2017b, 2019). The MTE, DPE and bridge elements are exclusively dependent on the presence of a functional initiator with a strict spacing requirement, and are typically enriched in TATA-less promoters (Danino et al., 2015; Vo Ngoc et al., 2019). The MTE and DPE are likely bound by TAF1, TAF2, and TAF7 subunits of TFIID (Verrijzer et al., 1995; Louder et al., 2016; Patel et al., 2020).

The partially overlapping elements (MTE, DPE and bridge motifs) were discovered *in Drosophila* and characterized independently, and the exact interplay between them was originally not resolved. Bioinformatic examination of the core promoter composition revealed that functional DPE motifs are typically accompanied by the bridge motif (Shir-Shapira et al., 2019). The transcriptional output of Fushi tarazu (Ftz) target genes is mostly dependent on the DPE motif, with contribution of the bridge I motif. We therefore envision bridge I as an “auxiliary” element, which supports the function of DPE-dependent transcription but not sufficient for fully restoring it upon DPE loss. Potentially, this could promote robustness, where a strong DPE accompanied by a bridge motif ensures proper docking of TFIID for accurate transcriptional activity. Notably, sequence biases in positions + 17, + 19, and + 24 within DPE promoters were reported (Kutach and Kadonaga, 2000; Arnold et al., 2017), suggesting that additional downstream nucleotide positions could contribute to transcriptional output. A recent comprehensive analysis using machine learning resulted in the identification of a 19 bp DPE-like stretch, termed downstream promoter region (DPR), which contributes to the transcriptional output of many human promoters (Vo Ngoc et al., 2020). Taken together, we propose to relate to the downstream core promoter region as a single transcriptional unit comprised of distinct promoter elements. The DPE motif might serve as the heart of this regulatory hub that helps recruit TFIID downstream of the TSS.

## The DPE Motif is Associated With Developmental Programs in *Drosophila*

Core promoter composition is not a passive component of the DNA, but rather an active regulatory module of transcriptional output (Juven-Gershon et al., 2006; Zabidi et al., 2015). A genome-wide comparison of promoter activity throughout embryogenesis in 5 *Drosophila* species spanning 25–50 million years of evolution, indicated that distinct core promoter elements are associated with different developmental times. Maternally expressed oocyte genes are enriched for DRE and Ohler-1/5/6/7 motifs, whereas zygotic genes expressed through embryogenesis are enriched for Inr, MTE and DPE motifs; larval-related transcripts are enriched for TATA box elements (Batut and Gingeras, 2017). These observations highlight the role of specific core promoter motifs to comprise an additional regulatory dimension of the expression pattern required throughout different developmental stages.

**FIGURE 2:**
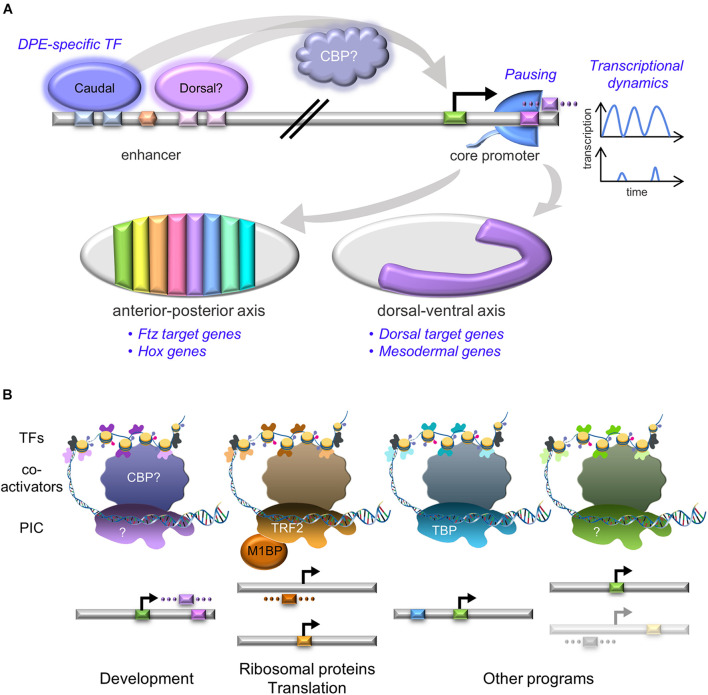
Unique core promoter assemblies are associated with diverse transcriptional programs. **(A)** DPE-containing core promoters are enriched in developmental programs. DPE-specific transcription factors (e.g., Caudal and perhaps Dorsal) are bound to enhancers, preferentially activating DPE-containing promoters. This regulation might be mediated by the CBP co-activator. Pol II promoter-proximal pausing (Pol II cartoon, blue) and transcriptional dynamics (blue graphs) comprise additional regulatory levels of developmental promoters. The graphs represent fluorescently-measured transcription intensities used to model bursting. All the above regulatory layers are then integrated to accomplish the developmental-related transcriptional program, resulting in gene expression that governs both anterior-posterior and dorsal-ventral axes of the developing *Drosophila* embryo, depicted as gray ovals (traditionally oriented with anterior to the left and dorsal side facing up). Left- stages 13–14 embryo, colored stripes represent distinct segment identity, based on specific Hox gene expression. Right- stage 8 embryo, purple region corresponds to mesoderm. CBP- CREB-binding protein, Ftz- Fushi Tarazu. **(B)** Distinct core promoter elements are associated with specific transcriptional programs. The core promoter elements are color-coded as the background colors in Figure 1. The faded core promoter represents promoters containing putative, yet undiscovered motifs. Transcription is initiated by the binding of the pre-initiation complex (PIC) to core promoter DNA. The identity of the PIC components likely differs between the various transcriptional programs, resembling the compatibility of each promoter type with the relevant basal transcription factors. Co-activators mediate PIC recruitment following binding of transcription factors (TFs) to enhancer regions, which contain binding sites for sequence-specific TFs. Some of these TFs may preferentially activate promoters containing specific core promoter elements (colored according to the core promoter composition). The diagram is not to scale. TBP- TATA box-binding protein; TRF2- TBP-related factor 2; M1BP- motif 1 binding protein; CBP- CREB-binding protein. DNA framework was modified from an image of the National Human Genome Research Institute Image Gallery.

The DPE motif was implicated in the control of developmental gene regulatory networks (GRNs) (Lenhard et al., 2012; Zehavi et al., 2014b), specifically during anterior-posterior and dorsal-ventral axes formation, including in mesoderm development in *Drosophila melanogaster* (Figure 2A). The homeotic (Hox) genes specify segment identity along the anterior-posterior axis of the developing embryo in all multicellular animals. All of the *Drosophila* Hox gene promoters lack TATA box elements, and the majority of them contain functional DPE motifs (Juven-Gershon et al., 2008). Importantly, the DPE is necessary *in vivo* for transcriptional regulation of the *Antp* P2 promoter within the developing *Drosophila melanogaster* embryo (Zehavi et al., 2014b). The *Drosophila* dorsal-ventral developmental GRN includes multiple genes that are activated by different nuclear concentrations of the Dorsal transcription factor along the dorsal-ventral axis. This GRN is dependent on the presence of the DPE motif; over two-thirds of Dorsal target genes contain DPE sequence motifs, which is significantly higher than the proportion of DPE-containing promoters in the genome. Multiple Dorsal target genes are evolutionarily conserved and functionally dependent on the DPE (Zehavi et al., 2014a). Furthermore, the observed expression levels of hybrid enhancer-promoter constructs recapitulates the levels detected for the relevant core promoter, and not the enhancer (Zehavi et al., 2014b). *fushi tarazu (ftz)* is a pair-rule gene orchestrating the segmentation phase of *Drosophila* embryonic development, expressed along the anterior-posterior axis (reviewed in Gehring and Hiromi, 1986). Ftz target genes are enriched for functional Inr + bridge + DPE combinations, conserved within *Drosophila* species (Shir-Shapira et al., 2019). Using CRISPR/Cas9, the *in vivo* contribution of the DPE motif to the regulation of the endogenous *tinman* gene in the developing *Drosophila* embryo was recently tested. Following mutation of *tinman*’s endogenous DPE motif using a co-CRISPR strategy, the endogenous *tinman* RNA levels were reduced twofold at 2–4 h of embryonic development (Levi et al., 2020). While the *in vivo* compatibility of distinct enhancers to their cognate promoters was previously demonstrated to determine expression levels during *Drosophila* development (Li and Noll, 1994; Merli et al., 1996), this is the first evidence for the contribution of a specific core promoter motif to transcriptional regulation of an endogenous gene. Furthermore, bioinformatics analysis revealed that *Drosophila* DPE-containing promoters are enriched for GO terms associated with embryonic development of the heart, circulatory system, skeletal muscle, peripheral nervous system, digestive system, renal system and reproductive system (Sloutskin et al., 2015). Thus, we propose that the DPE acronym, originally defined based on its downstream position, may be indicative of its function in *Drosophila* as a **D**evelopmental core **P**romoter **E**lement.

## Pol II Promoter-Proximal Pausing

The core promoter not only plays a role in Pol II recruitment to initiate transcription, it also regulates Pol II promoter-proximal pausing following transcription initiation. Pausing of transcriptionally engaged Pol II when the transcript is ∼25 nucleotides long was discovered in the *Drosophila heat shock protein 70* (*hsp70*) promoter (Gilmour and Lis, 1986; Rougvie and Lis, 1988). The development and application of genome-wide techniques directly measuring nascent RNA revealed that pausing of Pol II when the transcript is 20–60 nucleotides long is a common feature of *Drosophila* and mammalian genes under tight temporal control (reviewed in Core and Adelman, 2019; Lis, 2019; Wissink et al., 2019). In general, pausing extent and timing are regulated via nucleosome positioning (Gilchrist et al., 2010; Luse et al., 2020). Pol II pausing is now considered a major step regulating promoters that are required to elicit precise and rapid transcription, such as developmental and signal-responsive processes (reviewed in Gaertner and Zeitlinger, 2014; Mayer et al., 2017; Core and Adelman, 2019).

Pol II pausing is a major regulator of *Drosophila* morphogenesis, coordinating the synchronization of gene expression that is crucial for proper mesoderm development (Lagha et al., 2013). Analysis of core promoters of paused genes revealed that they are GC rich, and one-fourth of them contain a pause button (PB) motif (Hendrix et al., 2008). The PB motif consensus sequence “KCGRWCG” is enriched in the downstream core promoter, from + 1 to + 60 relative to the TSS. Moreover, the PB closely resembles the DPE, and can be located in close proximity to it. The exact interplay between the DPE and the PB, which is not strictly positioned relative to the Inr, awaits further investigation. GAGA factor binding sites and Ohler motif 1, as well as the transcription factors that specifically interact with them, also play a role in the regulation of pausing (Li and Gilmour, 2013). The precise mapping of Pol II on nascent RNA using PRO-seq demonstrated that the strength and position of sequence elements directly regulate pausing levels (Kwak et al., 2013). ChIP-nexus analysis demonstrated that downstream promoter sequences contribute to pausing stability, and a G at the + 2 position has an important role in stabilizing Pol II pausing (Shao et al., 2019). Remarkably, TFIID itself is required for establishing Pol II pausing, and depletion of its TAF1 and TAF2 subunits, but not of TBP, resulted in increased Pol II pause release and transcription re-initiation (Fant et al., 2020). Notably, some developmental promoters are paused, yet are not expressed in specific tissues (Gaertner et al., 2012), suggesting the involvement of other, tissue-specific, transcription factors in their regulation. These findings highlight the potential contribution of the downstream core promoter region to Pol II pausing, a critical regulatory aspect of transcriptional and developmental gene regulation.

## Diversity in TAFs and TBP Family Members is Associated With Different Core Promoter Compositions and Developmental Programs

Similar to core promoter sequence diversity, PIC composition is not universal, but rather promoter-dependent (illustrated in Figure 2B; Sikorski and Buratowski, 2009). This diversity is manifested by distinct TFIID complexes that contain paralogous TBP and tissue-specific TAF variants, a feature shared across metazoan organisms (Davidson, 2003; Freiman, 2009; Goodrich and Tjian, 2010; Ohler and Wassarman, 2010; Jones, 2014; Levine et al., 2014; Gupta et al., 2017). Depletion of TBP, TBP-related factor 2 (TRF2) and other TAFs using RNAi in *Drosophila* demonstrated the importance of TAF9, TAF5, and TRF2 to establish neural stem cell identity, emphasizing the role of specialized basal transcription machinery in developmental gene regulation (Neves and Eisenman, 2019). Tissue-specific complexes can also facilitate the discovery of additional core promoter elements. Indeed, the analysis of cell type-specific transcriptional programs during *Drosophila* spermatogenesis revealed the enrichment of novel downstream core promoter sequences bound by the spermatocyte-specific tMAC complex (Lu et al., 2020).

Such precise expression and function of tissue-specific TAF subunits is seen in diverse aspects of mammalian differentiation. *Taf4b*, *Taf7l*, and *Taf9b* are coordinately expressed in a highly germ cell-specific manner during mouse embryonic germ cell development (Gura et al., 2020). Interestingly, TAF7l and TAF9b are also required for regulating mouse adipocyte and neuronal differentiation, respectively (Zhou et al., 2014; Herrera et al., 2014). Selective expression and use of subsets of TAFs establishes a transcriptional program required for human embryonic stem cell (ESCs) self-renewal (Maston et al., 2012). Knockdown of multiple TFIID subunits affects the pluripotent circuitry in mouse ESCs and inhibits reprogramming of fibroblasts. Conversely, ectopic expression of mouse TAF4 greatly enhanced the reprogramming efficiency of mouse embryonic fibroblasts to induced pluripotent stem cells (Pijnappel et al., 2013). These data highlight the adaptability of selective assemblies of TAFs and their core promoter target sequences as key hubs for driving pluripotency and differentiation across highly divergent organisms.

The basal transcription factor TBP-related factor 2 (TRF2) is essential for embryonic development of *C. elegans*, *D. melanogaster*, *D. rerio*, and *X. laevis* (Dantonel et al., 2000; Kaltenbach et al., 2000; Veenstra et al., 2000; Muller et al., 2001; Kopytova et al., 2006). While TRF2 does not bind TATA-box-containing promoters, TRF2-containing complexes specifically interact with DPE-containing promoters and regulate TCT-containing ribosomal protein genes, among other gene classes (Bashirullah et al., 2007; Isogai et al., 2007; Oyama et al., 2013; Kedmi et al., 2014; Wang et al., 2014). Interestingly, M1BP was shown to interact with TRF2 and to regulate the expression of ribosomal protein genes (Baumann and Gilmour, 2017).

TRF2 emerged from TBP via gene duplication in the last common ancestor before the evolutionary split between bilaterians and non-bilaterian species (Duttke et al., 2014). Since the TRF2 protein lacks 3 of the 4 phenylalanine residues required for TATA box-binding, it may have evolved to support transcriptional programs dependent on TATA-less promoters, which are involved in the generation of the three germ layers (endoderm, mesoderm and ectoderm). Notably, DPE-containing genes are highly enriched for GO terms associated with embryonic development and particularly in mesoderm formation (Zehavi et al., 2014a; Sloutskin et al., 2015), suggesting that TRF2-based transcriptional programs might support mesoderm formation via the DPE. Moreover, TATA box and DPE-containing promoters can be regulated in an antagonist manner through TBP and NC2 with Mot1 binding, respectively (Hsu et al., 2008; van Werven et al., 2008). Interestingly, multiple *Xenopus* genes involved in mesoderm and organizer specification are TBP family-insensitive (Gazdag et al., 2016). The existence of multiple TBP family members with distinct characteristics, and of TBP family-insensitive developmental programs, are additional manifestations of the diversity in the transcriptional programs that support embryonic development through the downstream core promoter.

## Core Promoter-Enhancer Specificity, Similarity and Transcriptional Dynamics

While for many years it was thought that the specificity of gene expression is provided by enhancers, it is now clear that the core promoter region plays a key role in regulating gene- and cell type-specific transcription (Zehavi et al., 2014a; Zabidi and Stark, 2016). Distinct enhancers that preferentially work with either DPE- or TATA box-dependent *Drosophila* promoters have been identified (Butler and Kadonaga, 2001). Using a *Drosophila* cell culture-based reporter assay, developmental gene promoters were activated to higher levels than housekeeping gene promoters, with the Inr and DPE motifs contributing toward greater enhancer responsiveness than the TATA box (Arnold et al., 2017). Different TFs can have specific preferences for binding and activating promoters, based on their core promoter composition (Haberle et al., 2019). It was recently shown that the tMAC spermatocyte-specific complex binds novel promoter-proximal sequence motifs and opens the local chromatin to promote transcription initiation from downstream alternative promoters, indicating that proximal promoter composition may influence progression of differentiation programs as robustly as enhancers (Lu et al., 2020).

*Drosophila* Caudal, as well as mouse Caudal-related homeobox (Cdx) proteins (Cdx1, Cdx2, and Cdx4), which are key regulators of embryonic development and differentiation, activate transcription with a preference for a DPE relative to a TATA box (Juven-Gershon et al., 2008; Shir-Shapira et al., 2015). It is the unique combination of the *Drosophila* CREB-binding protein (dCBP) co-activator and Caudal that enables core promoter-preferential activation. Notably, dCBP co-occupies genomic loci in *Drosophila* embryos that are bound by Dorsal (Holmqvist et al., 2012; Holmqvist and Mannervik, 2013), in line with the involvement of CBP in Dorsal-target gene expression during embryogenesis (Akimaru et al., 1997) and with the enrichment of DPE motifs in mesodermal genes (Zehavi et al., 2014a). Tethering nuclease deficient Cas9 (dCas9) fused to either the Synergistic Activation Mediator (SAM) or the CBP HAT domain to the same promoters resulted in different activation levels for different promoters, supporting the concept of activator-promoter compatibility (Sajwan and Mannervik, 2019).

There is a large body of evidence supporting the similarity between promoters and enhancers, as many enhancers are transcribed and basal transcription machinery components are recruited to enhancers (Koch et al., 2011; Andersson et al., 2014; Core et al., 2014; Scruggs et al., 2015). Transcription of non-coding enhancer RNAs (eRNAs) is a common phenomenon discovered in human cell lines, correlated with the corresponding promoter’s strength (reviewed in Andersson et al., 2015). Both the existence of eRNA and the correlation to transcriptional output was confirmed in *Drosophila* embryos as well (Mikhaylichenko et al., 2018). The similarity between promoters and enhancers is reviewed in detail in Andersson and Sandelin (2020). Furthermore, core promoter sequences are necessary for proper enhancer function in human K562 cells, and can be used to predict and modulate enhancer activity (Tippens et al., 2020). A systematic dissection of distinct core promoter elements that function within transcribed enhancers will be instrumental.

High-precision methodologies enabled the examination of transcriptional dynamics, which demonstrated the existence of transcriptional bursts (Suter et al., 2011; Bothma et al., 2014). Burst size and initiation are controlled by the release of the paused Pol II molecules, while the recruitment rate remains the same across different promoters (Bartman et al., 2019; Cao et al., 2020). Both core promoter and TFIID compositions were shown to affect the magnitude of the initial transcriptional burst following the induction of cultured *Drosophila* cells with Cu (Pennington et al., 2013). The combination of advanced live-imaging techniques with quantitative analysis of transcriptional strength in *Drosophila* embryos revealed that enhancers control burst frequency (Fukaya et al., 2016). Interestingly, it was recently suggested that enhancer-promoter distance contributes to the regulation of gene activity by changing the size and the timing of transcriptional bursting in the developing *Drosophila* embryo (Yokoshi et al., 2020). Transcriptional dynamics is dictated not only by enhancers, but also by core promoter composition. Different core promoter sequences display divergent transcriptional dynamics profiles, observed in *Drosophila* embryos using MS2-based reporters (Fukaya et al., 2016). The TATA box and Inr elements affect burst size in mouse embryonic stem cells and in human fibroblasts (Larsson et al., 2019), whereas burst frequency is subject to regulation by enhancers and activators (Larsson et al., 2019; Wang Y. et al., 2020). The effects of endogenous core promoter motifs, including downstream elements, on transcriptional bursting remain to be determined.

## Concluding Remarks

We envision the core promoter composition as an additional regulatory dimension of the complex developmental GRNs. The DPE acronym, originally defined based on its position, may be indicative of its function as a **D**evelopmental core **P**romoter **E**lement. Notably, the downstream core promoter region is transcribed, thus one could speculate that it may exert a regulatory effect at the RNA level. The downstream core promoter region is an important regulatory tier of gene expression, likely conserved and diversified among metazoans. Thus, identification and characterization of downstream motifs in additional mammalian species would likely uncover the regulatory roles of mammalian downstream core promoters and their protein binding factors. Differential core promoter composition may provide a nuanced regulatory switch that recruits the relevant basal transcription factors at the right time during embryonic development to more precisely control the timing of developmental transcriptional programs. Being able to directly visualize and measure transcriptional dynamics will uncover novel insights regarding the functions, mechanisms and definitions of promoters and enhancers, which regulate the complex process known as “transcription.”

## Author Contributions

TJ-G and RF conceptualized the mini-review. AS and HS-S wrote the first draft of the manuscript. AS prepared the figures. AS and TJ-G finalized the review with input from all authors.

## Conflict of Interest

The authors declare that the research was conducted in the absence of any commercial or financial relationships that could be construed as a potential conflict of interest.

## Publisher’s Note

All claims expressed in this article are solely those of the authors and do not necessarily represent those of their affiliated organizations, or those of the publisher, the editors and the reviewers. Any product that may be evaluated in this article, or claim that may be made by its manufacturer, is not guaranteed or endorsed by the publisher.
